# Flexible Nylon Retractors: From Pupil Expansion to Capsule Support, an Analysis and Description of the Required Modifications to the Surgical Technique

**DOI:** 10.7759/cureus.99360

**Published:** 2025-12-16

**Authors:** Shahmeer H Noori, Diya Baker, Nick Kopsachilis, Gianluca Carifi

**Affiliations:** 1 Medical Education, Hillingdon Hospital, Uxbridge, GBR; 2 Ophthalmology, East Kent Hospitals NHS Foundation Trust, Canterbury, GBR; 3 Ophthalmology, University of Cambridge, Cambridge, GBR; 4 Ophthalmology, Ospedale Sanremo, Azienda Sanitaria Locale 1 (ASL1) Liguria, Sanremo, ITA; 5 Ophthalmology, Moorfields Eye Hospital, London, GBR

**Keywords:** capsular retractors, capsule hooks, capsule stabilization, capsule support, nylon retractors, phacodonesis, unstable lens, zonular instability, zonular weakness

## Abstract

In this report, we describe the technical modifications required for the safe and effective use of flexible nylon retractors for capsular support during phacoemulsification cataract surgery in cases of zonular weakness.

This study presents an analysis of surgical adaptations that allow conventional flexible nylon “iris” retractors to be repurposed for capsular stabilization. The key modifications include altering the geometry of the retractor tip from a 180° hook to an open 120° angle, optimizing incision placement and architecture, and standardizing retractor tensioning to minimize capsular stress. Practical steps are outlined for rhexis engagement, incision positioning, and tension control to maintain capsular bag centration and reduce intraoperative complications.

The modified technique was applied in a consecutive series of 28 eyes with varying degrees of zonular instability. No intraoperative anterior capsule tears or retractor slippages occurred following the introduction of the modified hook geometry, contrasting with previous experience using unmodified hooks. The modified approach provided stable capsular support throughout phacoemulsification, enabling controlled nucleus disassembly and intraocular lens implantation. Postoperative refractive outcomes were favorable, with 61% of eyes achieving a final refraction within 1.0 diopters of the target.

We believe that flexible nylon retractors, when appropriately modified, represent a safe, practical, and cost-effective option for temporary capsular support in the presence of zonular weakness. The described modifications, particularly the reshaping of the distal hook and strategic incision placement, distribute forces more evenly along the capsulorhexis edge, reducing the risk of slippage or capsule tearing. These refinements enhance intraoperative stability and offer an effective alternative in settings where specialized capsule support devices are unavailable. Further comparative studies are warranted to validate these findings and quantify long-term outcomes.

## Introduction

Management of subluxated cataracts presents surgical challenges. With recent advances in equipment and instrumentation, a judicious combination of surgical techniques, and a better understanding of fluid dynamics, the anterior segment surgeon should be able to perform a safe and successful phacoemulsification cataract extraction in the vast majority of cases presenting with compromised zonules. Currently, there is a host of devices available to help surgeons manage cases of weak zonules [1], with the use of flexible nylon retractors (commonly called “iris hooks”) suggested more than two decades ago for capsular stabilization [2].

These are readily available in most operating suites and familiar to most surgeons, as they are routinely employed for pupil expansion in cases of ineffective or insufficient preoperative pharmacological mydriasis [3] or for the prevention of intraoperative floppy iris syndrome and lens-iris diaphragm retropulsion syndrome [4-6].

Despite the above, the techniques outlined for the use of flexible retractors in cases of zonular instability remain variable. With the increasing popularity of specifically designed devices for zonular instability, such as capsular support hooks and tension segments, the role of iris hooks has come into question with concerns regarding possible intraoperative complications [7]. Nevertheless, iris hooks remain a practical and readily accessible alternative in settings where specialized devices are unavailable. Therefore, reinforcing the principles for safe and effective use is essential to ensure favorable surgical outcomes.

The present article aims to analyze the necessary changes to the surgical technique and to the geometry of the distal end of the device in order to guarantee reliable and reproducible use of common flexible nylon retractors for capsule support.

## Technical report

Modified geometry of the retractors

A fundamental modification that we suggest regards the geometry of the distal end of the device.

**Figure 1:**
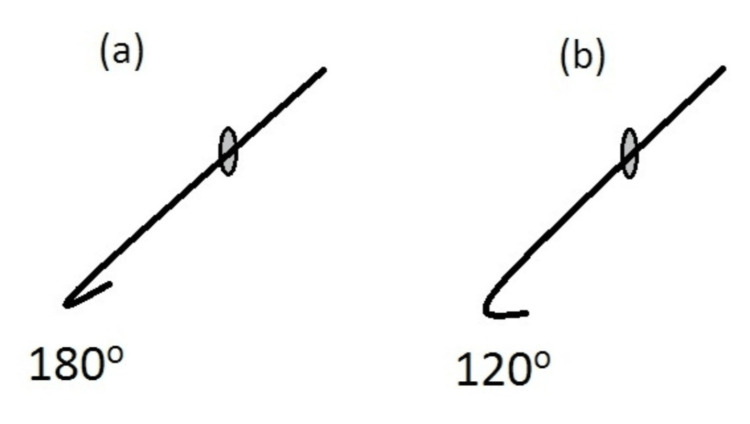
Necessary Modifications to Distal Geometry of the Retractor (A) The distal end of the flexible disposable polypropylene retractors is bent like a “U” and therefore resembles a hook. (B) The geometry of the distal end should be modified in order to increase the radius of curvature and achieve a bend of approximately 120 degrees.

While the intraocular end of the flexible retractors available on the market resembles a hook, with an angle of 180 degrees, we manually open the hook in order to obtain an angle of approximately 120 degrees (Figure 1).

This is easily performed with the use of two tying forceps, under the operating microscope, before inserting the hook into the corneal incision.

**Figure 2:**
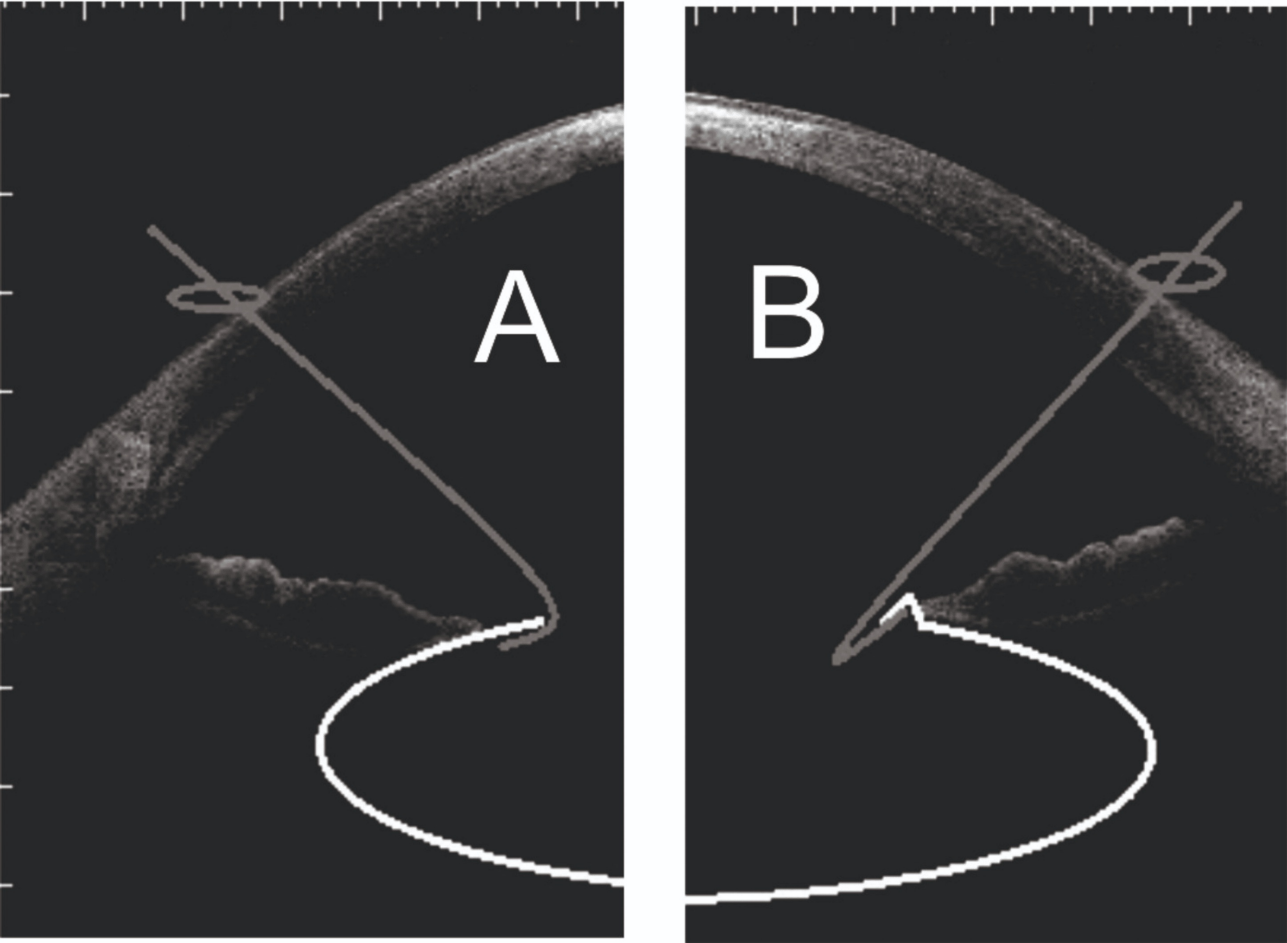
Modified Retractor vs Non-Modified Retractor Engagement of the Capsulorhexis Margin (A) With the modified geometry of the distal end, the open branch of the hook of the flexible retractor is placed flat under the edge of the capsulorhexis. (B) The non-modified hook applies forces on a tiny pressure point and determines folding of the capsulorhexis edge; this favors the slippage of the hook or, worse, the anterior capsule tearing during surgery

With the un-modified 180-degree angle hook, engagement of the capsulorhexis edge is possible, but its ability to provide consistent lift and stabilization of the anterior capsule is limited. This is because of the tight U-shaped curvature, which results in a small point of surface contact with the rhexis margin, increasing the risk of tenting or slipping (Figure 2). Opening the hook up to 120 degrees broadens the contact surface area, allowing the distal limb to lie parallel to the capsulorhexis margin, improving traction and distributing force more evenly. Thus, this modified design enhances the mechanical reliability and stability of iris hooks used in this context.

Size of the capsulorhexis

While a larger capsulorhexis (6-7 mm diameter) has been suggested in cases of zonular instability, we advise maintaining the normal rhexis size of about 5 mm. This would avoid the need for very steep corneal incisions, though it would conversely not facilitate the expression of the whole nucleus in the anterior chamber. Therefore, the surgeon must be confident that he will be able to disassemble the endo-nucleus within the capsular bag, at least in two hemi-nuclei.

Engagement of the rhexis

To facilitate a clear and net engagement of the capsular edge, we suggest performing a minimal anterior hydrodissection, followed by anterior viscodissection. Then, the balanced salt solution, the minimal amount of ophthalmic viscosurgical device (OVD) placed under the anterior capsule, will keep the rhexis edge slightly elevated above the lens material, and hooking the capsulorhexis edge becomes a simple task. A complete hydrodissection will then follow the final placement and tensioning of the four hooks.

Required tension

The forces applied to the edge of the capsulorhexis must be gentle to avoid further stress on the already compromised zonules. Once all hook-ends of the flexible retractors have been positioned into the anterior chamber and behind the capsulorhexis edge, a two-step tensioning process is carried out. The hooks are first retracted to gently engage the anterior capsule opening. We suggest retracting the two hooks on the same meridian before acting on the other pair. Once this is complete, a further retraction of the distal end of the hooks confers the required amount of tension to the capsule, and we suggest again retracting the hooks in pairs.

The appropriate final tension will square the capsule opening without pulling on the remaining zonules and must allow a residual elasticity to avoid anterior capsule tears during the subsequent surgical maneuvers.

The necessary retraction will mainly depend on the elasticity of the capsule: in young patients affected with collagen diseases such as Marfans disease, a greater degree of tension may be applied, although extensive intraocular and intracapsular manipulations is not expected; in very elderly patients, or when vision blue has been used, the more fragile status of the anterior capsule and the lower elasticity would advise toward the application of a more gentle tension.

Incision architecture, size, and location

**Figure 3:**
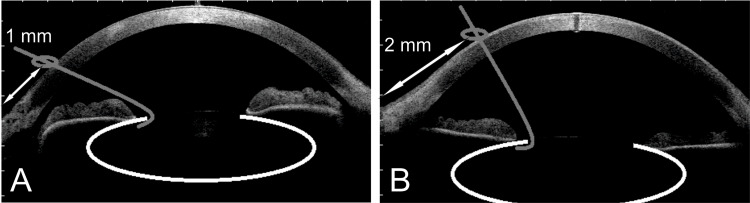
Stab Incision Positioning in Shallow vs Deep Anterior Chambers (A) Given that the length of the incision and the geometry of the distal end are not modified, the placement of the incisions in eyes with shallow anterior chambers is less advanced in clear cornea, (B) compared to eyes with deep anterior chambers.

We prefer to make the stab incisions after performing the continuous curvilinear capsulorhexis (CCC), as there is a clear reference point (the edge of the rhexis), and advise maintaining the same length. Hence, the incisions could also be advanced one or even 2 mm in clear cornea, based on the peripheral anterior chamber depth (Figure 3). Therefore, the incisions result in a steeper angle than those normally employed when iris hooks are needed, and this indication contrasts with previous recommendations of inserting the hooks posteriorly to the limbus [8], which is instead normally performed for iris hooks placement.

These aspects might have an effect on wound sealing in the immediate post-operative period. While it is important to achieve a self-sealing incision, the regularity of the internal wound profile might also be important, with a slit knife being therefore preferable to a microvitreoretinal blade.

**Figure 4:**
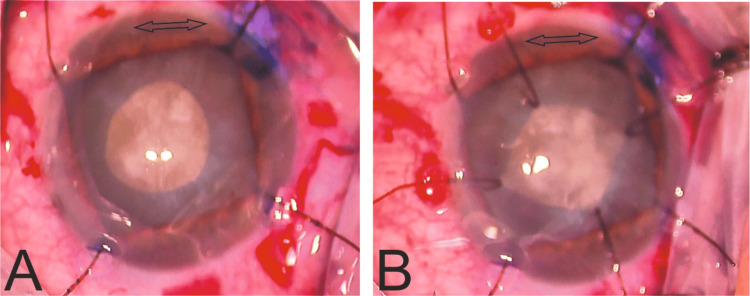
Orientation of Capsular Hooks Relative to the Main Incision Intraoperative images demonstrating the orientation of hook placement relative to the main incision to optimize capsular stability during phacoemulsification. (A) Two opposite stab incisions are positioned such that the proximal side of the squared rhexis lies parallel to the main incision (arrow), minimizing stress on the capsular edge during instrument entry. (B) Circumferential hook placement at 45° to the main incision allows for even distribution of traction forces and avoids alignment of rhexis corners with the main wound, thereby reducing the risk of anterior capsule tears.

We create the four stab incisions using a slit 15-degree corneal micro-blade, aiming for a width of 0.7 mm (23 G), approximately. Each opposite pair of incisions is placed on mutually perpendicular meridians. We suggest placing the incisions in a way that the two meridians result in an angle of 45 degrees from the main incision, to obtain the proximal side of the squared rhexis parallel to the entry wound, and perpendicular to the phaco needle. As a result, neither of the angles of the squared rhexis should be lying facing the main incision (Figure 4). This differs from the positioning of the hooks for pupil expansion, where we try to have one of the diagonals of the squared pupil aligned as much as possible with the meridian of the main incision, to obtain the best possible view along the action line where phaco-needle and chop mainly work.

## Discussion

Inadequate zonular support increases the risk of capsule complications during cataract extraction, presenting a number of surgical challenges [1]. Often, marked zonular instability is identified at the time of the preoperative ocular examination, allowing for proper planning of the surgical intervention, from the type of anesthesia to the placement of the required corneal incisions, and allows the surgical team to have the tools considered more effective in ensuring adequate intraoperative capsule support in the specific case available in the operating theatre.

Sometimes, the anterior segment surgeon faces challenging cataract removals in eyes with unforeseen and unexpected markedly compromised zonules. In fact, a marked preoperative pupil miosis, particularly in the case of posterior synechiae, might make less obvious the presence of a phacodonesis at the slit-lamp biomicroscopy, which can also be unrecognized due to the inexperience of more junior colleagues or trainees who might have performed the preoperative examination. In this respect, we favor the patient examination on the day of surgery by the operating surgeon, particularly in cases that could be associated with zonular problems. These include patients with a positive history of ophthalmic trauma, hereditary systemic disorder, or uveitis; patients previously undergone pars plana vitrectomy or any other intraocular surgical procedure; patients affected with high myopia or pseudoexfoliation syndrome; and elderly patients presenting with advanced cataracts.

First clues of unexpected zonular abnormalities manifest at the time of anterior chamber fill with ophthalmic viscosurgical device (OVD), as the anterior chamber might drastically deepen or might present with asymmetric depth, or most importantly at the time of capsulorhexis: incising the capsule with the cystotome might be more troublesome, or the peripheral capsular rim may tend to move along with the flap as it tears, due to lack of circumferential zonular tension. In such cases of unexpected zonular problems, the surgeon must rapidly consider whether expanding the equator of the capsular bag is sufficient to safely continue surgery, or whether he is facing a case of marked and generalised zonular impairment. In this regard, capsule tension rings redistribute the capsule forces across the remaining intact regions of zonules, and are optimally used if there is a focal and not marked zonular instability. The age of the patient also plays a role in this decision-making process, as generalized and progressive zonulopathy can lead to long-term dislocation of the capsule-intraocular lens complex, even if the capsule tension ring has been inserted [9].

The use of the so-called “iris hooks” should guarantee that the rhexis and the whole lens-capsule complex are kept centered, the antero-posterior excursions of the complex are avoided, and the torque of the bagis prevented or limited. In these extreme and challenging situations, the rotation of nuclear material should be limited or avoided, and the adoption of the step-by-step chop in-situ technique preferred [10].

The flexible nylon iris retractors are, or should be, always available in any surgical setup where cataract surgery is being performed, and surgeons are generally comfortable with the required surgical technique, from making the limbal incisions to the engagement of the pupillary edge.

Employing hooks in cases of unexpected zonular problems has unquestionable advantages and can be carried out at any time during the surgical procedure. For many surgeons who commonly operate under topical anesthesia, no change in anesthetic technique is needed, in contrast to other and more advanced devices for capsule stabilization, which often require transcleral sutures. In addition, there is a minimal loss of time, as they only require four corneal stab incisions and minimal intraocular maneuvering. The technical ability required to insert the hooks and to engage the capsule edge is essentially the same as for the use of iris retractors, with the only differences related to the incision location and the slightly different architecture, as described and summarized in Table 1.

**Table 1:** Iris Hooks Versus Modified Capsule Hooks*: the Required Modifications The table presents several technical differences necessary for a safe and effective use of the nylon retractors for capsule support. *5/0 nylon retractors. Thicker retractors might be difficult to model and might require larger incisions.

	Iris Hooks	Modified Capsule Hooks
Geometry of the distal end (Figure 1)	180 angle resembles a "U"	120 angle, circa resembles a closed "L."
Entry incision (Figure 3)	Limbus or posterior to limbus	1 or 2 mm advanced in the clear cornea
Location of incision	1 of the 2 meridians close to the main incision	Both meridians are 45 degrees away from the main incision
Incision width	0.6 mm circa	0.7-0.8 mm
Wound profile	Nearly parallel to the iris	With a steeper angle
Hook tensioning	Maximal to confer rigidity to the iris stroma and maximal dilation	Appropriate to square the rhexis edge while guaranteeing sufficient residual capsule elasticity

Although we describe a distal hook angle of approximately 120 degrees, this value is not intended to represent a precise or optimised angle. This modification was empirically derived in order to ensure the distal limb of the hooks lies flat beneath the capsulorhexis edge, mimicking the action of purpose-built capsule hooks. This approach promotes even distribution of tension across the larger contact surface area, minimizing the risk of excessive stress upon the capsule from point-loading that would occur with the original 180-degree angle (Figure 2).

The aforementioned details mean that the learning curve needed for a safe use of capsular hooks is very short when the modifications to the surgical technique we discuss are followed. We disagree with Bloom and Lee, who indicated the need for a great tension [11], and we consider it unsafe to markedly stretch the anterior capsulorhexis. This could lead to intraoperative tearing of the rhexis, with an easy and rapid extension of the tear to the posterior capsule in a contest of marked loss of zonular fibers, which would otherwise help contain such extension.

We also do not favor the adoption of six or even eight hooks as previously described [12], while sharing the idea that a fragile capsule, as observed in pseudoexfoliation, could be more prone to tearing. Dr Lavin’s suggestion is based on the concept that the forces applied during surgery on the capsule opening edge can be distributed on several contact points in a small area, with increased resistance to tearing. We believe instead that the correct answer to this problem lies in the modification of the geometry of the hook-end and in the variable advancement in clear cornea of the entry incisions, so that the forces are distributed along the contact area between the capsule and the open branch of the hooks, which will be lying flat and parallel underneath its edge, as shown in Figure 2. Furthermore, the modifications we proposed should also enhance the stabilization of the lens-capsule complex against the antero-posterior movement while limiting the tenting effect that could be produced on the capsulorhexis edge. These would prevent the slippage of the hooks over the capsule, which would otherwise need frequent repositioning during surgery or worse, as nucleus dislocation could occur when capsule stabilization is temporarily lost.

Although several techniques have been outlined for capsular stabilization in zonular weakness, the choice of support device remains a critical determinant of both intraoperative stability and long-term outcomes. Understanding where our approach fits amongst its counterparts is crucial for efficacious surgical planning.

A range of alternative devices is available to provide both intraoperative and long-term capsular support in the setting of zonular weakness. Purpose-made capsular support hooks function similarly to the modified iris hook, offering rapid segmental stabilization of the capsular bag, which is advantageous in unexpected zonular dialysis [7]. In contrast, the Capsular tension ring (CTR) can be employed in cases requiring circumferential equatorial support in mild-moderate zonular instability, usually when the area of weakness spans approximately 2-5 clock hours. For more extensive zonulopathy (five to seven clock hours), the Capsular tension segment (CTS) offers segmental support and can be anchored to the scleral wall for additional stability [13]. These devices, however, often necessitate prolonged operating time and additional instrumentation, including the use of iris or capsular hooks to facilitate insertion. [1, 14] 

Ceylan et al. compared the use of capsular support hooks to iris hooks, given their similar use in focal zonular weakness. They found no difference in post-operative visual outcomes but observed a higher complication rate with iris hooks. This included anterior capsule tears seen in four of the twenty eyes from the iris hook group, compared to none seen in the capsular support hook group [7]. Whilst capsular support hooks have been demonstrated to be safe and effective, their higher unit cost compared to disposable iris hooks may limit their availability in certain operative settings. Furthermore, it is worth noting that the technique we propose differs from that used in the comparative study. Although a similar stab incision approach is described, we modify the angle of the iris hook tip and deploy the hooks circumferentially as opposed to only at focal areas of zonular weakness. As described earlier, this approach distributes tension more evenly along the capsulorexhis edge, potentially reducing the risk of anterior capsule tears. By optimizing our surgical technique, we hope to narrow the gap between the dedicated capsular hooks and standard iris hook,s supporting favourable outcomes independent of resource availability.

Finally, while Santoro et al have proposed a combined use of capsule retractors and capsule tension ring to add equatorial support [15], we would also like to remind that appropriate use of OVDs and fluidics settings can greatly enhance the safety and reduce the risk of capsule complications during the steps of cataract surgery.

## Conclusions

In conclusion, the use of modified hooks with a view to supporting the capsular bag during phacoemulsification cataract surgery should be considered safe and effective. Since modifying our technique, we have never experienced an anterior capsule tear or a slippage of the hooks. In contrast, slippage of the hooks was fairly common previously, when the geometry was not manually altered. We ought to highlight that a conclusive evidence on whether the modifications we suggest guarantee better intraoperative results and enhance safety is not likely to be investigated: in fact, it is not possible to evaluate the elasticity of the anterior capsule in the different patients, nor it is possible to determine the degrees of zonular weakness that would be graded differently by different surgeons, making it therefore impossible to obtain a homogeneous population to study. Despite these limitations, the described modification offers a simple, reproducible adjustment that may help improve surgical stability in challenging cataract cases.
